# Redundancy as a Graph-Based Index of Frequency Specific MEG Functional Connectivity

**DOI:** 10.1155/2012/207305

**Published:** 2012-10-16

**Authors:** Claudia Di Lanzo, Laura Marzetti, Filippo Zappasodi, Fabrizio De Vico Fallani, Vittorio Pizzella

**Affiliations:** ^1^Department of Neuroscience and Imaging, “G. d'Annunzio” University of Chieti-Pescara, Via dei Vestini 31, 66100 Chieti, Italy; ^2^Institute of Advanced Biomedical Technologies, “G. d'Annunzio” University Foundation, Via dei Vestini 31, 66100 Chieti, Italy; ^3^Neuroelectrical Imaging and BCI Lab, IRCCS Fondazione Santa Lucia, Via Ardeatina, 306, 00179 Rome, Italy; ^4^Department of Physiology and Pharmacology, Sapienza University of Rome, Piazzale Aldo Moro 5, 00185 Rome, Italy

## Abstract

We used a recently proposed graph index to investigate connectivity redundancy in resting state MEG recordings. Usually, brain network analyses consider indexes linked to the shortest paths between cerebral regions. However, important information might be lost about alternative trails by neglecting longer pathways. We measured the redundancy of the connectivity by considering the multiple paths at the global level (i.e., scalar redundancy), across different path lengths (i.e., vector redundancy), and between node pairs (i.e., matrix redundancy). We applied this approach to a robust frequency domain functional connectivity measure, the corrected imaginary part of coherence. The redundancy in the MEG networks, for each frequency band, was significantly (*P* < 0.05) higher than in the random graphs, thus, confirming a natural tendency of the brain to present multiple interaction pathways between different specialized areas. Notably, this difference was more evident and localized among the channels covering the parietooccipital areas in the alpha range of MEG oscillations (7.5–13 Hz), as expected in the resting state conditions. Interestingly enough, the results obtained with the redundancy indexes were poorly correlated with those obtained using shortest paths only, and more sensitive with respect to those obtained by considering walk-based indexes.

## 1. Introduction

Over the last decade, there has been a growing interest in the detection of functional connectivity in the brain from different neuroelectromagnetic and hemodynamic signals recorded by several neuroimaging techniques. Many methods have been proposed and discussed in the literature with the aim of estimating the functional relationships among different cerebral structures [1, 2]. The recent application of graph theoretical analysis to human brain time series is a valuable approach to the study of functional brain architecture [3]. Graph theoretical properties of neural networks have been studied in healthy subjects [4–8] and in patients with brain pathologies such as Alzheimer's disease (AD) [9, 10], schizophrenia [11, 12], and brain tumors [13]. These studies suggest that brain functional network parameters might serve as useful biomarkers for neurocognitive disorders and to improve therapies [14]. 

Usually, graph-based studies consider indexes linked to the shortest path between two interacting cerebral regions. However, the consideration of the shortest pathway alone seems rather reductive, possibly providing incomplete information about the alternative longer pathways by which two cerebral regions could interact. In particular, in this work we considered *path*-based alternative longer pathways, that is, pathways in which nodes can be visited only once. Other strategies including nodes and links that can be revisited several times along the way (*walks*), as described in [15, 16] and named “communicability,” are possible although less biologically plausible.

The information about longer pathways appears strictly related to the concepts of “redundancy” and “robustness.” These properties are critical for the survival of many biological systems as they allow for reliable functions despite the death of individual elements. Indeed, the number of different pathways between two areas, rather than the shortest one, could highlight the importance of the link between two regions. Even more importantly, in brain pathologies (Alzheimer, Parkinson, Stroke, etc.) the availability of alternative anatomical and functional pathways would allow the brain to reshape its physiologic mechanisms in order to compensate the critical consequences of the disease [17].

A graph-based index that includes robustness—taking inspiration from the recent findings on the evaluation of multiple paths between network elements [18–20]—has been defined and successfully applied in rest EEG [21]. In particular, three complementary indexes have been derived: (i) the scalar redundancy, a scalar number that characterizes the global level of redundancy in the network; (ii) the vector redundancy, a vector characterizing the overall redundancy for each path length; (iii) the matrix redundancy, a matrix describing the redundancy in each of the node pairs regardless of path length.

Indeed, De Vico Fallani and colleagues [21] showed that functional brain networks exhibit a high degree of redundancy, consistently with a natural tendency of the brain to establish multiple connections. However, EEG connectivity profiles are known to be strongly biased from volume conduction effect due to the low pass spatial filtering properties of the head and are influenced by the choice of the reference electrode [22, 23].

In this work, we calculate redundancy indexes from rest MEG data to avoid possible confounds from volume conduction and reference electrode effects. In fact, MEG does not require a reference channel and is intrinsically less influenced by volume conduction effect [24]. Moreover, the connectivity metric estimated from MEG data is a corrected version of the imaginary part of coherency [25] aimed at suppressing a spatial bias towards remote interactions. Finally, redundancy indexes from rest MEG data are compared to those identified using shortest path and walk-based pathways.

## 2. Materials and Methods

### 2.1. MEG Recordings

The present data were acquired in 7 healthy young adult subjects. The study was approved by the local ethical committee and all subjects gave their written informed consent. The subjects contributed one 5 min resting state MEG run during which they were instructed to maintain fixation on a visual crosshair. MEG was recorded using the 165-channel MEG system installed at the University of Chieti [26]. This system includes 153 dc SQUID integrated magnetometers arranged on a helmet covering the whole head plus 12 reference channels. Two electrical channels were simultaneously recorded (electrocardiogram and electrooculogram) to be used for artifact rejection. All signals were band-pass-filtered at 0.16–250 Hz and digitized at 1025 Hz. The position of the subject's head with respect to the sensors was determined by five coils placed on the scalp recorded before and after each MEG run. The coil positions were digitized by means of a 3D digitizer (3Space Fastrak; Polhemus), together with anatomical landmarks (left and right preauricular and nasion) defining the head coordinate system. After downsampling to 341 Hz, the recorded data were analyzed using Independent Components Analysis (ICA) by means of the fast ICA algorithm [27]. The ICs were automatically classified, the artefactual components were removed, and the nonartifactual ICs were then recombined thus providing cleaned time domain signals. In the present work, we considered only 61 evenly spaced MEG channels to compare the results with those reported with standard 64 ch EEG data [21]. 

### 2.2. Functional Connectivity

In the present study, we estimated a corrected version of the imaginary part of coherence, a robust measure of the linear relationship between two-time series in the frequency domain [25, 28]. 

Given two-time domain signals, *x*_*i*_(*t*) and *x*_*j*_(*t*), and their fourier transforms, *X*_*i*_(*f*) and *X*_*j*_(*f*), coherence is a complex valued measure of interaction defined as

(1) $$\begin{array}{c} C_{ij}\left(f\right)\equiv \frac{S_{ij}\left(f\right)}{\sqrt{S_{ii}\left(f\right)S_{jj}\left(f\right)}}, \end{array}$$

where

(2) $$\begin{array}{c} S_{ij}\left(f\right)\equiv \langle X_{i}\left(f\right)X_{j}^{*}\left(f\right)\rangle \end{array}$$

is the cross-spectrum between *X*_*i*_(*f*), and *X*_*j*_(*f*), *S*_*ii*_(*f*) is the power spectrum of *X*_*i*_(*f*), and *S*_*jj*_(*f*) is the power spectrum of *X*_*j*_(*f*). The symbols ∗ and 〈 〉 in (2) indicate complex conjugation and expectation value, respectively. In practice, expectation value is estimated as the average over signal epochs. 

A nonvanishing imaginary component of complex coherence (ImCoh) can only indicate a phase-shifted relationship between *X*_*i*_ and *X*_*j*_. As a consequence of this property, and assuming the quasistatic regime for Maxwell's equations, ImCoh is robust to self-connectivity induced by volume conduction or crosstalk at the sensor level [25, 29]. Thus, ImCoh robustly measures functional connectivity [30–33] meaning that a significant deviation from zero cannot be generated by independent sources but rather by true brain interaction. 

Since classical ImCoh might exhibit a spatial bias towards remote interactions, we rely on a corrected version of ImCoh (cImCoh) with the same properties introduced above and with the additional feature of compensating for preference for remote interactions [28]:

(3) cImCohij(f)≡Im⁡(Cij(f))(1−Re(Cij(f))2).

The corrected ImCoh was estimated for each run as the average over signal epochs of 2-second duration. Therefore, a single complex coherence value for each frequency bin was generated for each possible channel pair combination. In order to study the level of synchronization in specific physiological frequency bands, we averaged the corrected imaginary coherence values within specific ranges, thus, generating a single value for each frequency band of interest, namely: delta (1–3.5 Hz), theta (3.5–7.5 Hz), alpha (7.5–13 Hz), beta (13–24 Hz), and gamma (24–60 Hz). The frequency band specific values were thus, stored in a channel matrix. This matrix describes a functional network, where the particular combination of the *i*th row with the *j*th column indicates the synchronization value between the MEG signals of the *i*th and *j*th channels. At this stage, the functional brain connectivity is a fully connected and undirected network. To compute topological features, a network has to be converted into undirected and unweighted graph by considering a threshold, expressed as connection density, that represents the number of the most powerful connections to be considered. We choose an “optimal” connection density of 0.101, as this is the best statistical tradeoff to differentiate between the global and local structural properties of a network with 61 nodes. This highest separation would increase the independence between the two indexes when measuring the global and local properties of the network [34, 35]. This threshold retains the 370 highest values (in magnitude) for the MEG network by setting them equal 1 and by setting the remaining ones to 0.

### 2.3. Network Redundancy

**Figure 1:**
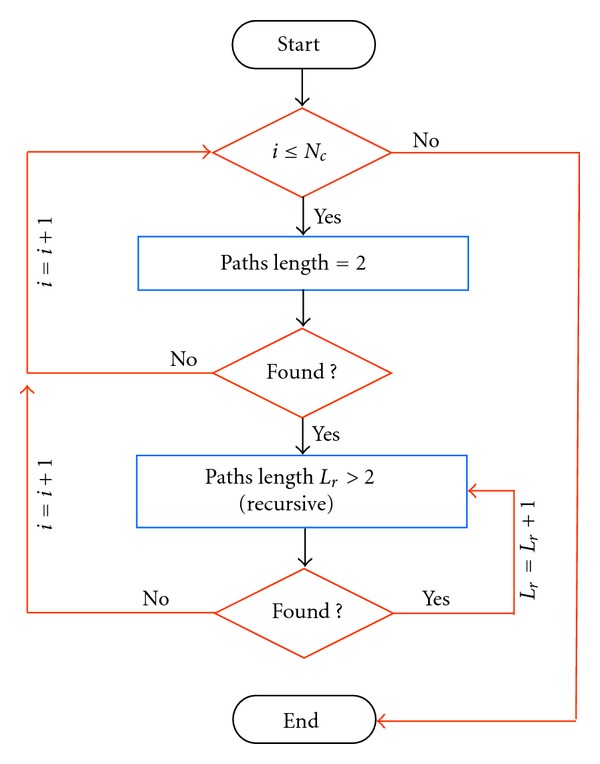
The main steps of this algorithm are highlighted in the flowchart that computes all the possible paths in a graph, able to count the total number of paths between the nodes excluding vertices already visited (self-connections).

A graph is defined as a set of vertices/nodes *N* and a set of links/connections representing some sort of interaction between the vertices. The adjacency matrix *A* of size *N* × *N* contains the information about the graph connectivity structure. If a link connects the two nodes *i* and *j*, the corresponding entry of *A* is given by *a*_*ij*_ = 1; otherwise, *a*_*ij*_ = 0. In a graph, a path is an alternating sequence of vertices and links, beginning and ending with a vertex, where each vertex is incident to both the preceding link and the following link in the sequence. Given such definition, it is clear that the shortest path is only one of the possible ways in which two nodes in a graph can interact. To account for all the possible ways, longer pathways should also be considered for characterizing functional brain connectivity [34, 35]. Our algorithm, implemented in Matlab (The MathWorks Inc., Natick, MA, USA), computes all the possible paths in a graph by counting the total number of links between the nodes excluding vertices already visited (self-connections). The main steps of this algorithm are highlighted in the flowchart of Figure 1. The algorithm output is a three-dimensional matrix *P* of size *N* × *N* × *L*, containing the number of all the possible paths of length *l* = 1,…, *L* in each node pair, where *L* ≤ *N* − 1. Starting from this *P*-matrix, we evaluate the following characteristic measures.

#### 2.3.1. Scalar Redundancy

The scalar redundancy *R*_*s*_ is the total sum of the number of paths, of any length *l* = 1,…, *N* − 1, found between all the nodes, that is, (*N* ^2^ − *N*)/2, excluding the self-connections:

(4) $$\begin{array}{c} R_{s}=\sum_{i=1}^{N}\sum_{j=1}^{N}\sum_{l=1}^{L}P\left(i,j,l\right). \end{array}$$

It represents the global level of network redundancy by means of a scalar number. The higher is *R*_*s*_, the higher is the tendency of the graph to exhibit multiple alternative pathways.

#### 2.3.2. Vector Redundancy

The vector redundancy *R*_*v*_ is the total sum of the number of paths found between all the nodes, that is, (*N* ^2^ − *N*)/2, excluding the self-connections, with respect to each path length *l* = 1,…, *N* − 1:

(5) $$\begin{array}{c} R_{v}\left(l\right)=\sum_{i=1}^{N}\sum_{j=1}^{N}P\left(i,j,l\right). \end{array}$$

It represents the total level of network redundancy across different path lengths. The higher is *R*_*v*_(*l*), the higher is the tendency of the graph to exhibit multiple alternative pathways with a specific length *l*.

#### 2.3.3. Matrix Redundancy

The matrix redundancy *R*_*m*_ is the total sum of the number of paths of any length *l* = 1,…, *N* − 1 in each node pair:

(6) $$\begin{array}{c} R_{m}\left(i,j\right)=\sum_{l=1}^{L}P\left(i,j,l\right). \end{array}$$

It represents the total level of redundancy between the nodes of the graph. The higher is *R*_*m*_(*i*, *j*), the higher is the tendency of the graph to exhibit many alternative pathways between the nodes *i* and *j*.

In the present study, the analysis of the network redundancy indexes was addressed by exploring paths of a maximal length of *L* = 5.

#### 2.3.4. Random Network Comparison

The same redundancy indexes were computed in a set of reference graphs whose links were arranged in a random fashion. Indeed, random connections correspond to a scrambled situation, where no anatomical nor functional organization is implied, and are a baseline for the evaluation of all networks. In this work, 100 random graphs were generated by maintaining the same number of nodes and connections of the original MEG networks. Each time, links were randomly shuffled without preserving the node degree distribution [36]. This choice is motivated by the fact that the networks are rather small (61 MEG channels) and sparse (connection density ~0.1), and preserving the degree distribution would generate very similar network topologies due to reduced number of different possible random combinations.

Finally, the statistical contrast with the random networks was addressed for the experimental subjects and for each frequency band by calculating the *z*-score of the obtained redundancy indexes. 

#### 2.3.5. Comparison with Other Indexes

Redundancy indexes were also compared with those found by using the shortest paths between all the node pairs. Starting from the three-dimensional matrix *P* of size *N* × *N* × *L* defined before, and containing the number of all the possible paths of length *l*, we calculated the matrix PS containing the number of shortest paths between the nodes (two-dimensional matrix of shortest path):

(7) $$\begin{array}{c} \text{PS}\left(i,j\right)=P\left(i,j,k\right),\\ k=\min\left(l\right)\;\text{such}\;\text{that}\;\;P\left(i,j,k\right)>0. \end{array}$$

Shortest path-based PS values were compared with the redundancy matrix index *R*_*m*_ (with *L*_max⁡_ = 5). In order to reduce any effect related to the different range of values (i.e., the number of shortest paths could significantly deviate from the number of paths of any length), the original values were normalized by the mean values obtained from 100 random networks through a *z*-score. Then, the difference was assessed by computing the difference of the normalized matricial values, for each subject and frequency band.

Similarly, we implemented a matricial index using the number of alternative pathways (with *L*_max⁡_ = 5) as revealed by walks, along the line of the communicability concept introduced in [16]. Starting from the adjacency matrix *A*, we evaluated the matrix communicability index *G* containing the number of walks of length *l* = 1,…, *L*_max⁡_ that started at node *i* and finished at node *j*:

(8) $$\begin{array}{c} G\left(i,j\right)=\sum_{l=1}^{L_{\max}}A^{l}\left(i,j\right). \end{array}$$

Again, *G*(*i*, *j*) was compared with the redundancy matrix index *R*_*m*_. In order to reduce any effect related to the different range of values (i.e., the number of walks could significantly deviate from the number of paths of any length), the original values were normalized by the mean values obtained from 100 random networks through a *z*-score. Then, the difference was assessed by computing the difference of the normalized matricial values, for each subject and frequency band.

## 3. Results

**Figure 2:**
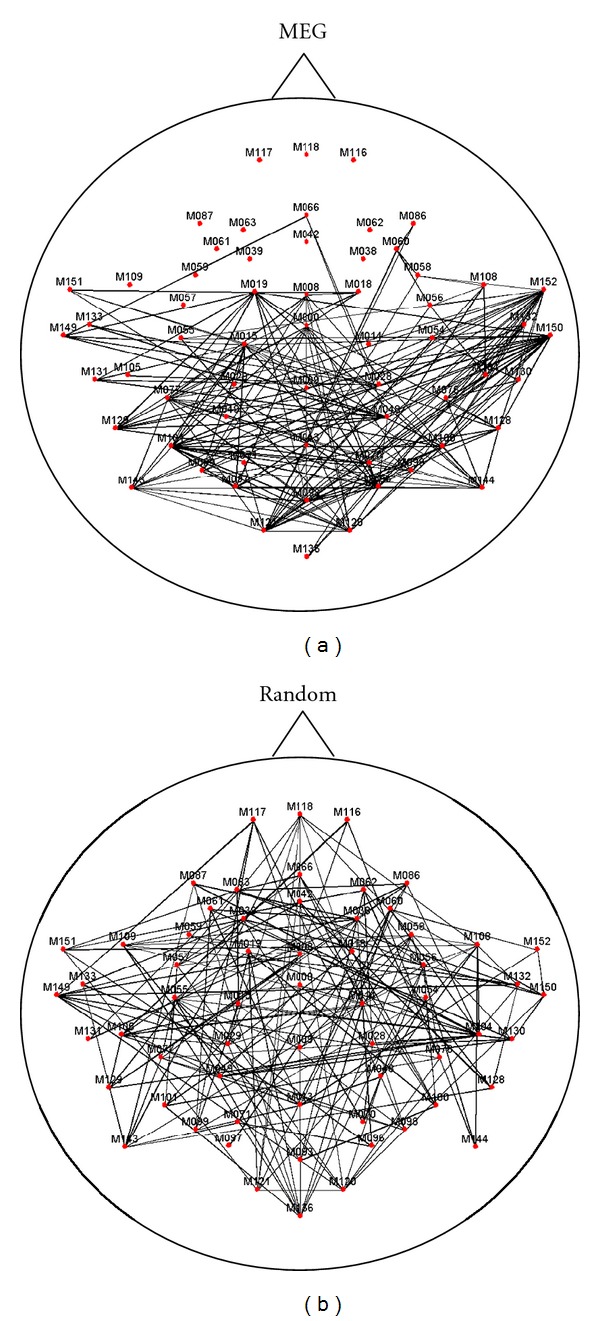
(a) Representation of the MEG network in alpha frequency band. Nodes represent MEG channels, while links indicate a significant synchronization in the frequency domain between the time series of all the MEG channels (1342 highest values of corrected imaginary coherence magnitude). (b) Representation of a simulated random network with same number of nodes and links of the MEG network.

The MEG network in the alpha frequency band relative to one subject is shown in Figure 2(a), whilst Figure 2(b) shows one random network obtained by randomizing the original links among the channels. As it can be observed, there is a clear difference between the two connectivity patterns. Notably, in the MEG network the nodes of the temporal, parietal, and occipital areas are strongly interconnected, while there is no particular structure in the random network. 

**Figure 3:**
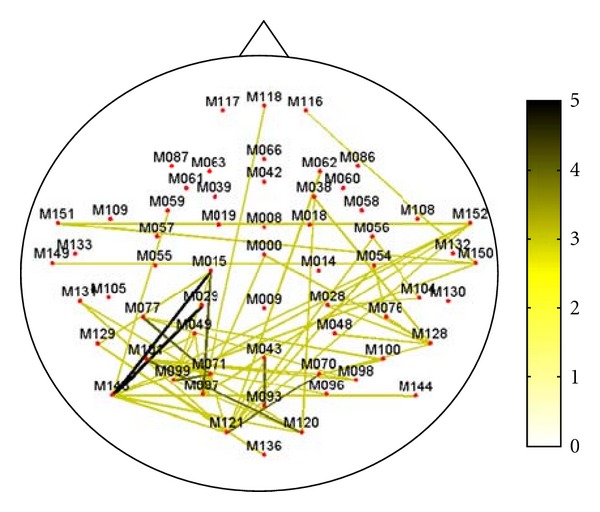
Representation of the MEG network in alpha frequency band for all subjects. Nodes represent MEG channels, while thickness and color line code the number of subjects who share that particular connection. Only values larger than 2 are shown. According to the color bar, connections common to many subjects are identified by green-black colors.

Figure 3 shows the cumulative MEG graph in alpha frequency band relative to all of the 7 subjects. 

Only values larger than 2 are shown. The cumulative MEG network resembles the functional structure shown in Figure 2(a), highlighting the consistency of temporal, parietal, and occipital interconnections in resting state MEG networks.

All three redundancy indexes—*R*_*s*_, *R*_*v*_, *R*_*m*_—computed for MEG data showed statistically significant difference (*P* < 0.05) with respect to the random graph set for all frequency bands: delta, theta, alpha, beta, and gamma, as indicated by *z*-scores listed in Table 1. 

**Figure 4:**
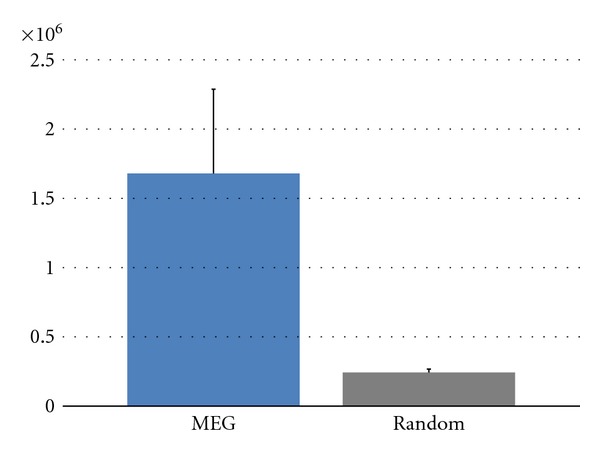
Mean scalar redundancy values for MEG network in the alpha frequency band and for random graphs. Vertical bars denote the standard deviation (of the values of 7 subjects for MEG graph and of the values of 100 random graph). A significant difference between the MEG and random values is found (*P* < 0.05).

**Figure 5:**
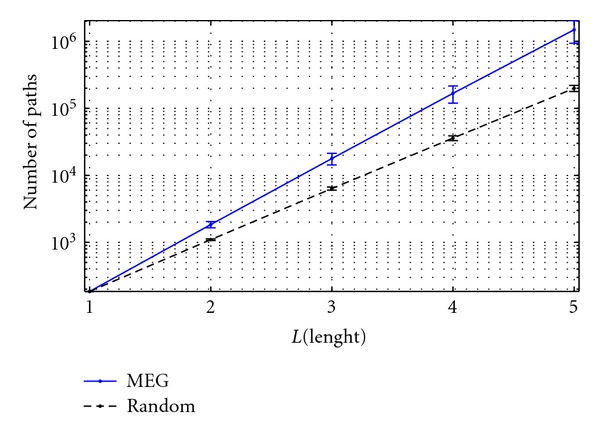
Mean vector redundancy values in logarithmic scale for MEG network in the alpha frequency band (solid blue line) and for random graphs (dashed black line). Vertical bars denote the standard deviations. A significant difference between the MEG and random values is found (*P* < 0.05).

Figure 4 details mean *R*_*s*_ values in the alpha frequency band calculated from single subject MEG networks as well as the mean *R*_*s*_ values from the random networks. The scalar redundancy in the MEG networks is significantly higher (*P* < 0.05) with respect to random graphs.  Figure 5 details mean *R*_*v*_ values in MEG and random networks in the alpha band. Although they have similar trends, showing a vector redundancy that increases with path length, the statistical comparison between their values is highly significant. In particular, the vector redundancy of the MEG network is significantly higher (*P* < 0.05) than that of random graphs for the path lengths *l* = 2,…, 5. Actually, the results for *l* = 1 are identical due to the statistical threshold that made all the inspected networks having the same number of connections (see Functional connectivity paragraph in Section 2). 

**Figure 6:**
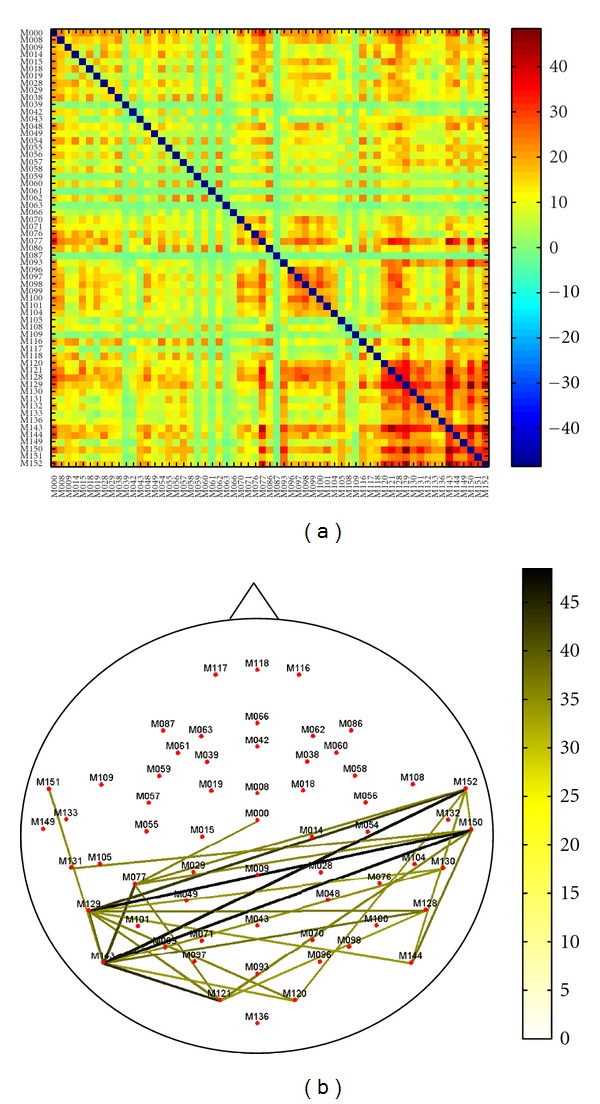
*z*-score values of matrix redundancy for MEG network in the alpha frequency band. (a) the degree of *z*-score redundancy for each channel pair is colour coded: highest values of redundancy, significantly different between MEG and random networks, are identified by yellow-red colours. (b) only values larger than 70% of the maximum value are shown. According to the colour bar, highest values of redundancy, significantly different between MEG and random networks, are identified by green-black colours.

Figure 6 shows the mean *z*-score values of the matrix redundancy *R*_*m*_ for the representative alpha frequency band. Also in this case, *R*_*m*_ calculated for MEG networks is significantly different from that of random graphs. Similar results were also obtained in the other frequency bands. Furthermore, MEG networks show a clear topographical specificity as revealed by a very high redundancy between the nodes of the parietal and occipital areas.

**Figure 7:**
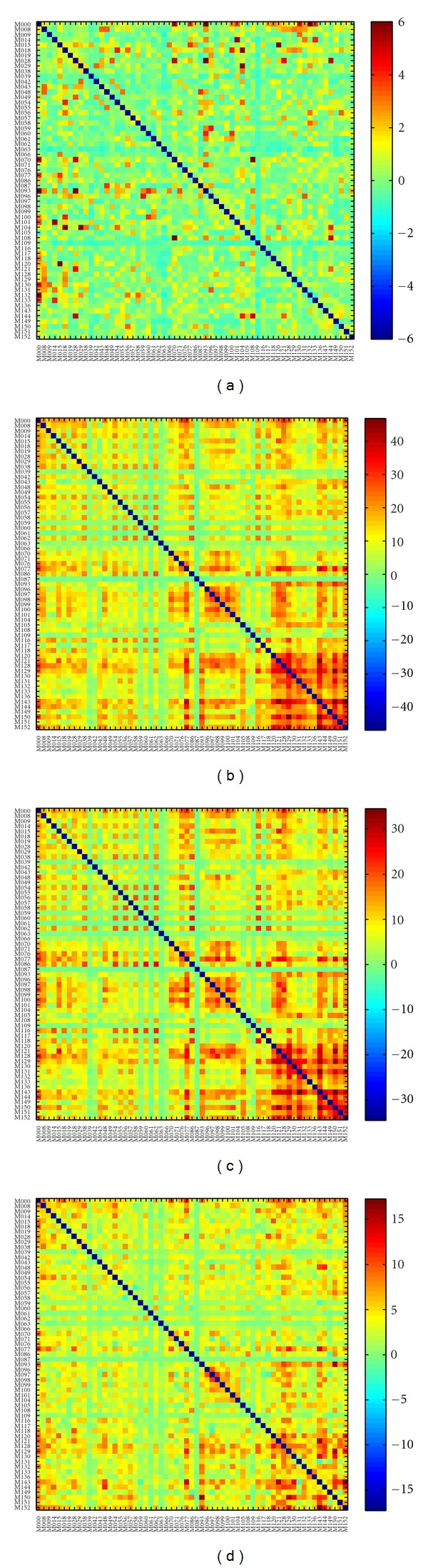
Comparison between redundancy, shortest path, and communicability for the alpha frequency band: (a) *z*-score values related to matrix containing the number of shortest paths between all the node pairs; (b) difference between mean *z*-score values of redundancy and mean *z*-score of shortest path; (c) *z*-score values of matrix of communicability, that is, redundancy indexes based on the computation of walks; (d) difference between mean *z*-score values of redundancy and mean *z*-score values of communicability.

Finally, Figure 7 summarizes the comparison of redundancy index with those obtained from shortest path and communicability in the alpha frequency band. In particular, we show in Figure 7(a) the mean *z*-score values related to shortest path-based matricial index, in Figure 7(c) the mean *z*-score values related to communicability-based matricial index. Figures 7(b) and 7(d) show the difference between the mean *z*-score values for the matrix redundancy index (shown in Figure 6(a)) and the mean *z*-score for the shortest path and the communicability, respectively. Moreover, the number of nodes significantly correlated according to Spearman coefficient between redundancy index and shortest path-based index is 0.93% (*P* < 0.05, corrected for multiple comparisons through the rough false discovery rate [37]), while is much higher (56,12%) between the redundancy index and communicability-based index. Similar results were obtained in the other frequency bands.

## 4. Discussion

In this study, we derived graph theory parameters from a robust frequency domain functional connectivity measure, the corrected imaginary part of coherence estimated from MEG data. Indeed, MEG is immune to reference electrode effect and is less confounded by volume conduction effect [24]. The graph connectivity structure is represented as a binary quantity in the adjacency matrix and provides information about the links between vertices (i.e., MEG channels). Our data show that the MEG network features a less spread topology with respect to similar networks mapped by EEG [21]. In fact, the widespread topology found in EEG can possibly be ascribed to volume conduction effect and/or to the bias towards remote interactions.

In our study, we calculated three different indexes: scalar, vector, and matrix, to the aim of characterizing overall network redundancy, global network redundancy for a given path length, and redundancy of pairwise connections in the network. As a general rule, these indexes are related to the maximum path length (*L*_max⁡_) explored. The results presented here are obtained for *L*_max⁡_ = 5, which corresponds to a computationally reasonable amount of time and space (20 s per subject and per frequency band on a Intel i5-2400 CPU @ 3.10 GHz with 8 GB of RAM). The needed amount of time diverges for higher *L*_max⁡_ values. Nevertheless, as it can be seen from Figure 5, where the dependence of vector redundancy from *L* is shown, a linear trend (in semilogarithmic scale) rules such dependence. Thus, the vector redundancy for higher values of *L*_max⁡_ can be extrapolated. Moreover, the matrix redundancy obtained for *L*_max⁡_ = 5 typically shows a high spatial correlation degree with respect to matrix redundancy obtained for higher *L* values (up to 10), meaning that topographical information is preserved also for lower *L* values. Graph theory parameters derived from adjacency matrices are usually calculated by considering the shortest possible pathway of interaction between two vertices. Nevertheless, shortest distances alone could provide an incomplete characterization of a network, since connectivity in complex systems with similar shortest paths distribution can indeed, exhibit distinct structural and dynamical properties [34, 35]. In particular, by neglecting the longer pathways important information might be lost about the alternative trails that could connect two nodes in a network. The possibility to inspect multiple pathways within a system is strictly related to the concept of redundancy and robustness, which is supposed to be a natural mechanism of the brain for enhancing the resilience to neural damages and dysfunctions [38].

Scalar redundancy is related to overall network resilience. This index appeared significantly different than the corresponding value obtained from random networks in all the frequency bands.

Similar results were obtained with EEG recordings [21]. This difference suggests that scalar redundancy might be a functional correlate of brain connectivity disruption with a possible prognostic value. 

Vector redundancy is related to global network redundancy for a given path length. Higher vector redundancy values for MEG graphs compared to random graphs indicate the network tendency to build a larger number of connections for a given path length *L*, regardless of specific node contribution. Analogous results were found for EEG [21]. Again, this parameter might serve as a prognostic index. 

The matrix redundancy index informs us about the robustness of a given pairwise connection. Indeed, our data showed the most redundant interactions between the parietal and occipital channels in the alpha frequency band, as expected in relation with the posterior alpha rhythm originating in occipito-parietal areas during rest [39]. Notably, our MEG results on the network topology show an improved spatial specificity with respect to its EEG counterpart [21], possibly thanks to the diminished bias from volume conduction and reference electrode effects.

**Methodological Considerations**

One of the main issues related to the used redundancy indexes is if they carry different information from other existing measures like for instance that related to the shortest paths or to walks.

As it can be noticed by comparing Figures 6 and 7(a), the connections characterized by high redundancy values differ from those obtained using shortest path-based values. A direct comparison between the *z*-scores (Figure 7(b)) shows that the redundancy values were generally higher than shortest path-based values, the difference being largely positive. Interestingly, the highest differences were located between the occipito-parietal regions. The significant correlation (Spearman) between the distribution of the shortest path-based and redundancy index gathered from the population observed in less than 1% of the connections strengthens the finding that the two indexes are not related and provide different information. Taken together these results indicate that the topological information carried by shortest paths is different from that obtained by redundancy. Furthermore, these two measures are generally not correlated, thus, justifying the additional time needed for redundancy computation.

When comparing communicability-based and redundancy matrix index, we observed a high degree of correlation. We would like to stress that the redundancy indexes are based on paths which never visit the same vertex twice, [40] thus, avoiding cycles that have a difficult interpretation in functional brain networks and that are generally neglected by the existing literature [41]. From a general point of view, this can be seen as the main difference between the present method and the communicability-based indexes. Indeed, pathways visiting a node more than once are fake alternatives to the possibly damaged link. To give an example, a link between two nodes (just suppose that this is the only way they can connect) is identified as a walk of distance equal to 3 and as a path of distance 1. In our view, there is no real redundant information between these two nodes, since they are directly connected as correctly identified by the path-based distance.

Nevertheless, Figure 7(d) shows that *z*-scores found from the redundancy values were generally higher than the ones obtained from communicability-based values. Thus, there exists a general tendency of the walk-based index to overestimate the number of actual interactions between nodes, and to generate lower *z*-scores with respect to redundancy values. This suggests that redundancy indexes are in general more sensible in identifying significant redundant interactions between nodes.

Overall, in the present work, we demonstrated that a natural high degree of redundancy, confidently ascribed to functional brain network behavior, is also exhibited by the MEG networks in a group of healthy subjects. Moreover, although we believe that it is not good practice to draw strong conclusions about the underlying brain functioning from channel level information, our results may be attributed to the role of alpha band in mediating interactions in or between visual, attention, and default mode networks [42].

Finally, it would be interesting to investigate how different mental states or behavioral conditions, as well as alterations due to cerebral diseases, can affect this high natural redundancy of spontaneous functional brain networks.

## 5. Conclusion

This work has shown that functional brain networks as measured by MEG exhibit a natural high redundant degree of frequency specific interaction between different regions. The redundancy indexes used are defined to capture different information at the global level (scalar), at each path length (vector), and between any node pair (matrix). In our opinion, this information might integrate rather than substitute indexes based on the shortest path, thus, allowing for a more comprehensive understanding of network properties. In particular, the inclusion of redundancy metrics in a benchmark set of graph indexes might be particularly relevant for studying plasticity in connectivity pattern organization [17] such as those occurring during brain development in the first span and in healthy aging [6] in the last part of life, as well as brain injuries or diseases (e.g., Alzheimer's disease, brain tumors, etc.) [38]. 

## Figures and Tables

**Table 1:** Mean *z*-score values of scalar redundancy (*R*_*s*_) and vector redundancy (R_v_). Different rows correspond to different bands. In the R_v_ section, each column refers to a different path length *l*. Asterisks denote a significant (*P* < 0.05) difference from random graphs.

	*R*_*s*_	*R*_*v*_				
		*l* = 1	*l* = 2	*l* = 3	*l* = 4	*l* = 5
Delta	38.32*	—	20.34*	23.74*	30.95*	39.60*
Theta	45.30*	—	19.86*	25.56*	34.41*	47.16*
Alpha	60.03*	—	24.77*	33.21*	45.76*	62.42*
Beta	39.40*	—	20.36*	24.62*	31.78*	40.72*
Gamma	54.54*	—	24.40*	31.41*	42.09*	56.68*

## References

[1] Baccalá LA, Sameshima K (2001). Partial directed coherence: a new concept in neural structure determination. *Biological Cybernetics*.

[2] Kuś R, Kamiński M, Blinowska KJ (2004). Determination of EEG activity propagation: pair-wise versus multichannel estimate. *IEEE Transactions on Biomedical Engineering*.

[3] Bullmore E, Sporns O (2009). Complex brain networks: graph theoretical analysis of structural and functional systems. *Nature Reviews Neuroscience*.

[4] Bassett DS, Meyer-Lindenberg A, Achard S, Duke T, Bullmore E (2006). Adaptive reconfiguration of fractal small-world human brain functional networks. *Proceedings of the National Academy of Sciences of the United States of America*.

[5] Micheloyannis S, Pachou E, Stam CJ, Vourkas M, Erimaki S, Tsirka V (2006). Using graph theoretical analysis of multi channel EEG to evaluate the neural efficiency hypothesis. *Neuroscience Letters*.

[6] Micheloyannis S, Vourkas M, Tsirka V, Karakonstantaki E, Kanatsouli K, Stam CJ (2009). The influence of ageing on complex brain networks: a graph theoretical analysis. *Human Brain Mapping*.

[7] Smit DJA, Stam CJ, Posthuma D, Boomsma DI, De Geus EJC (2008). Heritability of “small-world” networks in the brain: a graph theoretical analysis of resting-state EEG functional connectivity. *Human Brain Mapping*.

[8] Stam CJ (2004). Functional connectivity patterns of human magnetoencephalographic recordings: a “small-world” network?. *Neuroscience Letters*.

[9] Stam CJ, Jones BF, Nolte G, Breakspear M, Scheltens P (2007). Small-world networks and functional connectivity in Alzheimer’s disease. *Cerebral Cortex*.

[10] Stam CJ, De Haan W, Daffertshofer A (2009). Graph theoretical analysis of magnetoencephalographic functional connectivity in Alzheimer’s disease. *Brain*.

[11] Micheloyannis S, Pachou E, Stam CJ (2006). Small-world networks and disturbed functional connectivity in schizophrenia. *Schizophrenia Research*.

[12] Rubinov M, Knock SA, Stam CJ (2009). Small-world properties of nonlinear brain activity in schizophrenia. *Human Brain Mapping*.

[13] Bartolomei F, Bosma I, Klein M (2006). Disturbed functional connectivity in brain tumour patients: evaluation by graph analysis of synchronization matrices. *Clinical Neurophysiology*.

[14] Deuker L, Bullmore ET, Smith M (2009). Reproducibility of graph metrics of human brain functional networks. *NeuroImage*.

[15] Crofts JJ, Higham DJ, Bosnell R (2011). Network analysis detects changes in the contralesional hemisphere following stroke. *NeuroImage*.

[16] Estrada E, Hatano N (2008). Communicability in complex networks. *Physical Review E*.

[17] Duffau H (2006). Brain plasticity: from pathophysiological mechanisms to therapeutic applications. *Journal of Clinical Neuroscience*.

[18] Costa LF, Rodrigues FA Superedges: connecting structure and dynamics in complex networks.

[19] Rodrigues FA, da Fontoura Costa L (2009). A structure-dynamic approach to cortical organization: number of paths and accessibility. *Journal of Neuroscience Methods*.

[20] Rodrigues FA, Da Fontoura Costa L (2010). Generalized connectivity between any two nodes in a complex network. *Physical Review E*.

[21] De Vico Fallani F, Toppi J, Di Lanzo C (2012). Redundancy in functional brain connectivity from eeg recordings. *International Journal of Bifurcation and Chaos*.

[22] Marzetti L, Nolte G, Perrucci MG, Romani GL, Del Gratta C (2007). The use of standardized infinity reference in EEG coherency studies. *NeuroImage*.

[23] Nunez PL, Srinivasan R, Westdorp AF (1997). EEG coherency I: statistics, reference electrode, volume conduction, Laplacians, cortical imaging, and interpretation at multiple scales. *Electroencephalography and Clinical Neurophysiology*.

[24] Srinivasan R, Winter WR, Ding J, Nunez PL (2007). EEG and MEG coherence: measures of functional connectivity at distinct spatial scales of neocortical dynamics. *Journal of Neuroscience Methods*.

[25] Nolte G, Bai O, Wheaton L, Mari Z, Vorbach S, Hallett M (2004). Identifying true brain interaction from EEG data using the imaginary part of coherency. *Clinical Neurophysiology*.

[26] Della Penna S, Del Gratta C, Granata C (2000). Biomagnetic systems for clinical use. *Philosophical Magazine B*.

[27] Hyvarinen A, Karhunen J, Oja E (2001). *Independent Component Analysis*.

[28] Ewald A, Marzetti L, Zappasodi F, Meinecke FC, Nolte G (2012). Estimating true brain connectivity from EEG/MEG data invariant to linear and static transformations in sensor space. *NeuroImage*.

[29] Schoffelen JM, Gross J (2009). Source connectivity analysis with MEG and EEG. *Human Brain Mapping*.

[30] Guggisberg AG, Honma SM, Findlay AM (2008). Mapping functional connectivity in patients with brain lesions. *Annals of Neurology*.

[31] Martino J, Honma SM, Findlay AM (2011). Resting functional connectivity in patients with brain tumors in eloquent areas. *Annals of Neurology*.

[32] Marzetti L, Del Gratta C, Nolte G (2008). Understanding brain connectivity from EEG data by identifying systems composed of interacting sources. *NeuroImage*.

[33] Nolte G, Marzetti L, Valdes Sosa P (2009). Minimum Overlap Component Analysis (MOCA) of EEG/MEG data for more than two sources. *Journal of Neuroscience Methods*.

[34] De Vico Fallani F, Baluch F, Astolfi L, Subramanian D, Zouridakis G, Babiloni F (2010). Structural organization of functional networks from eeg signals during motor learning tasks. *International Journal of Bifurcation and Chaos*.

[35] De Vico Fallani F, Rodrigues FA, Da Fontoura Costa L (2011). Multiple pathways analysis of brain functional networks from eeg signals: an application to real data. *Brain Topography*.

[36] Sporns O, Zwi JD (2004). The small world of the cerebral cortex. *Neuroinformatics*.

[37] Benjamini Y, Yekutieli D (2001). The control of the false discovery rate in multiple testing under dependency. *Annals of Statistics*.

[38] Hallett M (2000). Brain redundancy: responsivity or plasticity?. *Annals of Neurology*.

[39] Goldman RI, Stern JM, Engel J, Cohen MS (2002). Simultaneous EEG and fMRI of the alpha rhythm. *NeuroReport*.

[40] Bollobas B (1998). *Modern Graph Theory*.

[41] Rubinov M, Sporns O (2010). Complex network measures of brain connectivity: uses and interpretations. *NeuroImage*.

[42] Mantini D, Perrucci MG, Del Gratta C, Romani GL, Corbetta M (2007). Electrophysiological signatures of resting state networks in the human brain. *Proceedings of the National Academy of Sciences of the United States of America*.

